# *Gallic*, *Aconitic*, and *Crocetin* Acids as Potential TNF Modulators: An Integrated Study Combining Molecular Docking, Dynamics Simulations, ADMET Profiling, and Gene Expression Analysis

**DOI:** 10.3390/molecules30153175

**Published:** 2025-07-29

**Authors:** Adolat Manakbayeva, Andrey Bogoyavlenskiy, Timur Kerimov, Igor Yershov, Pavel Alexyuk, Madina Alexyuk, Vladimir Berezin, Vyacheslav Dushenkov

**Affiliations:** 1 Research and Production Center for Microbiology and Virology, Almaty 050010, Kazakhstan; adolat.manakbayeva@mail.ru (A.M.);; 2 Department of Natural Sciences, Hostos Community College CUNY, Bronx, NY 10451, USA; vdushenkov@hostos.cuny.edu

**Keywords:** TNF, immunomodulator, molecular docking, ADMET, gene expression

## Abstract

Organic acids, as natural metabolites, play crucial roles in human metabolism and health. Tumor Necrosis Factor (TNF), a pivotal mediator in immune regulation and inflammation, is a key therapeutic target. We evaluated ten organic acids as TNF modulators using in silico molecular docking, followed by detailed ADMET (Absorption, Distribution, Metabolism, Excretion, and Toxicity) profiling and molecular dynamics (MD) simulations for three lead candidates: *gallic*, *aconitic*, and *crocetin* acids. Their effects on TNF gene expression were then assessed *in vivo* using a mouse leukocyte model. The in silico results indicated that *crocetin* had the highest TNF binding affinity (−5.6 to −4.6 kcal/mol), while *gallic* acid formed the most stable protein-ligand complex during MD simulations, and *aconitic* acid established hydrogen bond interactions. ADMET analysis suggested potential pharmacokinetic limitations, including low permeability. Contrasting its high predicted binding affinity, *in vivo* gene expression analysis revealed that *crocetin* stimulated TNF synthesis, whereas *gallic* and *aconitic* acids acted as inhibitors. This research explores organic acids as potential TNF modulators, highlighting their complex interactions and providing a foundation for developing these compounds as anti-inflammatory agents targeting TNF-mediated diseases.

## 1. Introduction

The synthesis and transformation of most compounds within complex biological systems often involve the formation of significant amounts of organic acids [1,2]. Studies have demonstrated that various organic acids play crucial roles in both human health and disease [3,4,5]. These compounds participate in diverse biological processes, including anti-inflammatory, immunoregulatory, anti-edematous, antidiabetic, anticancer, cardiovascular, hepatoprotective, and neuroprotective activities.

One of the key processes implicated in the onset and progression of numerous diseases is inflammation. Recent studies have shown that organic acids can attenuate inflammation by modulating several signaling pathways, reducing the production of pro-inflammatory mediators, and enhancing anti-inflammatory mediators. Research demonstrates that organic acids suppress cellular inflammation by downregulating interleukin IL-6 and reactive oxygen species (ROS) while upregulating IL-10 levels [6]. For instance, butyrate mitigates lipopolysaccharide (LPS)-induced inflammation by specifically upregulating IL-10, particularly under septic shock conditions [7].

Acetate mitigates neutrophilic inflammation by inducing caspase-dependent neutrophil apoptosis, suppressing NF-κB signaling, and enhancing the production of anti-inflammatory mediators (IL-10, TGF-β, and annexin A1) [8]. Notably, inverse correlations exist between organic acid concentrations and TNF-α levels. However, these compounds may demonstrate context-dependent, bidirectional immunomodulation. For instance, in multiple sclerosis patients, butyrate and valerate concentrations positively correlate with IFN-γ and TNF-α, whereas acetate shows an inverse relationship with IFN-γ [9].

Tumor necrosis factor (TNF) plays a pivotal role in immune regulation and inflammatory disease pathogenesis. While anti-TNF biologics have proven successful in treating conditions like psoriasis and rheumatoid arthritis, they face significant limitations including immunogenicity, adverse effects, and high costs [10,11]. This has prompted the exploration of alternative TNF modulation strategies, particularly small-molecule inhibitors and receptor-specific targeting. Preclinical studies demonstrate that receptor-selective approaches—such as TNFR1 antagonists and TNFR2 agonists—show particular promise compared to global TNF blockade [12,13].

Additionally, small-molecule modulators targeting TNF expression, processing, receptor binding, and direct inhibition are being developed as potential alternatives to oral biopharmaceuticals [14,15]. These novel approaches aim to provide safer, more effective, and cost-efficient treatments for TNF-mediated inflammatory and autoimmune disorders.

The COVID-19 pandemic caused by SARS-CoV-2 underscored the crucial role of inflammation in disease severity. A cytokine storm, characterized by excessive production of pro-inflammatory cytokines—particularly TNF-α, IL-6, and IL-1β—can trigger multiorgan damage and acute respiratory distress syndrome (ARDS) [16,17]. This hyperinflammatory state involves both innate immune system dysregulation and renin-angiotensin system dysfunction [17].

Targeting cytokine storms has emerged as a promising therapeutic strategy, with TNF inhibitors showing potential to reduce circulating pro-inflammatory cytokines and potentially mitigate disease progression [18]. Additional investigational approaches include JAK inhibitors, IL-6/IL-1 receptor antagonists, and immunomodulators [16,19]. An integrated treatment regimen combining anti-inflammatory agents with antivirals and anticoagulants may represent an optimal strategy for managing severe COVID-19 cases [19].

Drug discovery remains an exceptionally resource-intensive endeavor, typically requiring 12–15 years of development with costs reaching 2.6 billion USD per approved compound [20,21,22]. Conventional high-throughput screening methods evaluate thousands of candidates, yet only 0.003–0.01% ultimately gain clinical approval [21]. The process faces significant challenges, particularly in reliably predicting human pharmacokinetics, toxicity profiles, and therapeutic efficacy during preclinical stages [23].

To overcome these challenges, bioinformatics and artificial intelligence (AI) have emerged as indispensable tools in modern drug discovery. The EIIP/ISM bioinformatics platform accelerates lead compound identification and therapeutic target discovery [21]. AI and machine learning enable a comprehensive analysis of large datasets, uncovering biological mechanisms and streamlining novel compound development. These advanced technologies can significantly reduce both the time and cost of drug development [20,23].

Computational modeling has become an indispensable component of modern drug discovery and development, significantly improving efficiency and cost-effectiveness in early-stage research. Advanced techniques, including molecular docking, ADMET analysis, and molecular dynamics simulations, provide powerful tools for evaluating compound-target interactions and predicting pharmacological properties [23,24]. These approaches enable researchers to screen drug candidates, optimize molecular interactions, and identify promising lead compounds [23]. When integrated with machine learning algorithms, computational modeling substantially enhances predictive accuracy in drug discovery pipelines [25].

Despite persistent challenges such as scoring function limitations and the requirement for experimental validation, computational methods have become essential across multiple drug discovery domains, including formulation development, analytical research, and process chemistry [26]. Ongoing advancements in the field, particularly the integration of machine learning with molecular modeling, continue to enhance and refine drug discovery methodologies.

Tumor necrosis factor (TNF) plays a critical role in regulating inflammatory processes, immune responses, and cellular apoptosis [27,28,29,30]. Dysregulated TNF activity is associated with numerous autoimmune and inflammatory disorders, including psoriasis, inflammatory bowel disease, and rheumatoid arthritis. The identification of novel natural compounds capable of modulating TNF activity represents an important focus in contemporary biomedical research. In this study, we explore the potential of organic acids as TNF activity modulators.

## 2. Results

### 2.1. Computational Investigation of Organic Acids as Potential Tumor Necrosis Factor (TNF) Inhibitors by Molecular Docking

To evaluate organic acids as potential immunomodulators of tumor necrosis factor, molecular docking was performed on 10 selected organic acids. Two standard TNF inhibitors were utilized to validate the docking method employed. The binding affinities of these organic acids to the TNF protein ranged from −5.6 kcal/mol to −2.6 kcal/mol and are presented in Table 1.

Among the ten compounds tested, *gallic* acid, *aconitic* acid, and *crocetin* were selected for further analysis based on a combination of factors, including structural diversity, biological relevance, and favorable binding parameters. *Gallic* acid, a well-characterized phenolic compound known for its anti-inflammatory activity, was used as the reference molecule. *Aconitic* acid was chosen due to its tricarboxylic structure and capacity to form numerous hydrogen bonds, despite its moderate binding energy. *Crocetin* exhibited the highest affinity for TNF, as indicated by the molecular docking results.

It is important to note that the selection of compounds also took into account such criteria as substance availability, their toxicological safety according to the literature, and the potential for structural optimization. Although acids such as syringic and vanillic demonstrated comparable docking scores, they were not included in further study due to structural similarity to other compounds or the lack of distinct pharmacological features. Thus, the inclusion of chemically diverse compounds in the final analysis allowed us to obtain a more comprehensive understanding of the nature of ligand interactions with TNF within the framework of various structural features of organic acids.

To detail the specific interactions between the TNF protein and *gallic* acid, *aconitic* acid, and *crocetin*, including hydrogen bonds (H-bonds) and van der Waals forces, *LigPlot* was used to generate 2D visualizations for each compound Table 2.

Molecular docking of the interaction of *gallic*, *aconitic* acids, and *crocetin* showed that these compounds can suppress tumor necrosis factor by forming fairly stable complexes between the protein and the ligand.

### 2.2. Molecular Dynamics Simulation and System Stability Analysis

To validate the molecular docking results, molecular dynamics (MD) simulations were conducted to investigate the structural and dynamic characteristics of TNF complexes with *gallic* acid, *aconitic* acid, and *crocetin*. The trajectory analysis allowed for the evaluation of complex stability, protein flexibility, compactness, solvent accessibility, and hydrogen bonding interactions with the ligands.

**Figure 1:**
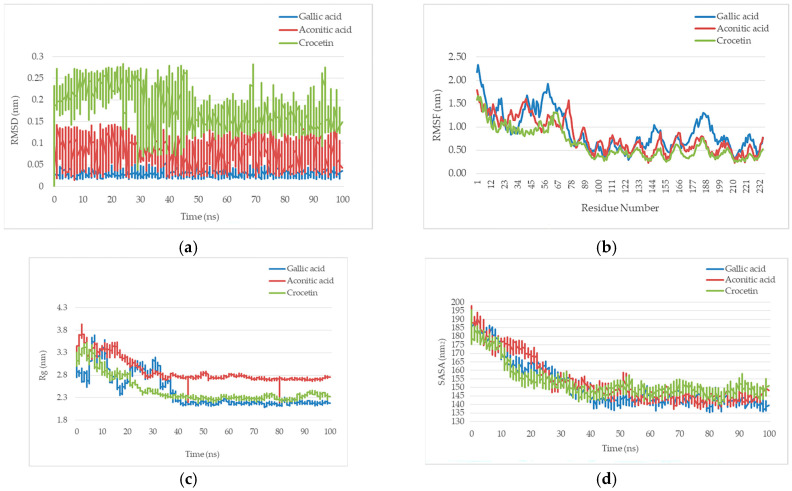
Molecular Dynamics Simulation of organic acids with TNF. (**a**) The root mean square deviation (RMSD) of the TNF protein in complexes with ligands over 100 ns of simulation. (**b**) Fluctuations of individual amino acid residues (RMSF) in TNF complexes with ligands. (**c**) Changes in the radius of gyration (Rg) of the TNF protein in complexes with ligands over 100 ns of simulation. (**d**) Solvent-accessible surface area (SASA) of the TNF protein in complexes with ligands over 100 ns of simulation.

The root mean square deviation (RMSD) analysis demonstrated that the TNF-*gallic* acid complex exhibited the highest structural stability throughout the simulation, with RMSD values remaining around 0.05 nm, indicating minimal conformational changes (Figure 1). The TNF-*aconitic* acid complex showed slightly greater RMSD fluctuations, reaching approximately 0.1 nm, yet maintaining an overall stable state. In contrast, the TNF-*crocetin* complex exhibited the largest structural deviations, with RMSD values reaching 0.25 nm, suggesting significant conformational changes upon ligand binding.

The root mean square fluctuation (RMSF) analysis of individual amino acid residues revealed that the highest flexibility was observed in the N- and C-terminal regions of the TNF protein, which is consistent with the expected structural characteristics (Figure 1). The average RMSF values for the central areas of the protein remained below 0.5 nm, indicating relative rigidity. Notably, the TNF-*gallic* acid complex exhibited the most pronounced fluctuations, suggesting its influence on local protein dynamics. Conversely, *crocetin* appeared to stabilize the central domains of TNF, reducing structural fluctuations.

Analysis of the radius of gyration (Rg) (Figure 1) showed an initial decrease in values for all complexes during the first 20 ns of the simulation, reflecting the stabilization of the protein structure after relaxation. Subsequently, the TNF complexes with *gallic* and *aconitic* acids showed minor fluctuations in Rg in the range of 2.6–2.8 nm. In contrast, the TNF-*crocetin* complex had the lowest and most stable Rg value (~2.3 nm), indicating a more compact conformation of the protein upon binding to *crocetin* compared to the other compounds.

A decrease in SASA values during the first 20 ns, followed by stabilization at 135–145 nm^2^, was clearly shown by the solvent-accessible surface area (SASA) analysis (Figure 1). This suggests a partial reduction in TNF solvent exposure upon ligand binding, potentially due to protein structural rearrangement. The TNF-*crocetin* complex exhibited higher SASA values, consistent with its higher Rg values, confirming a less compact TNF structure in this complex.

**Figure 2:**
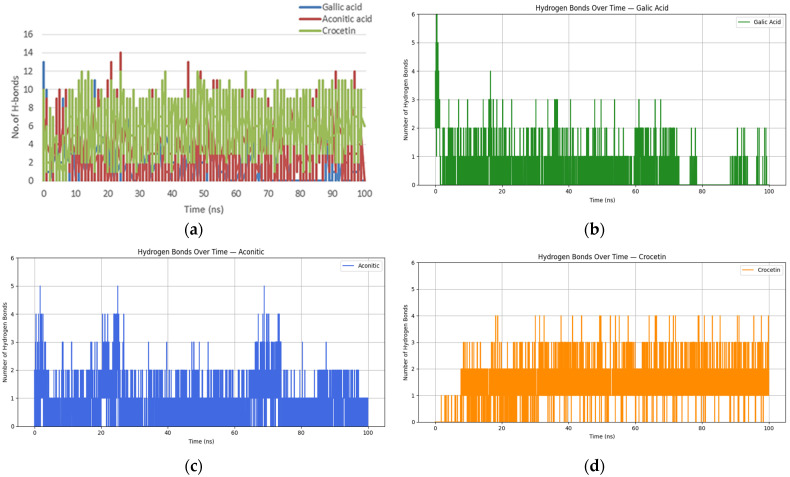
Number of hydrogen bonds formed between TNF and different ligands during the molecular dynamics simulation. (**a**) Comparative analysis of hydrogen bond formation in TNF complexes with *gallic* acid, *aconitic* acid, and *crocetin* over 100 ns. (**b**) Hydrogen bond dynamics in the TNF-*gallic* acid complex. (**c**) Hydrogen bond dynamics in the TNF-*aconitic* acid complex. (**d**) Hydrogen bond dynamics in the TNF-*crocetin* complex.

Hydrogen bond analysis revealed that the TNF-*aconitic* acid complex formed the highest number of hydrogen bonds, reaching up to 12 at certain time points, highlighting the role of hydrogen bonding in its stability (Figure 2). The TNF-*gallic* acid complex maintained between 4 and 8 hydrogen bonds, confirming its stable ligand-protein interactions (Figure 2b). The TNF-*crocetin* complex exhibited 6–10 hydrogen bonds, though with more pronounced fluctuations, indicating dynamic and transient interactions over time (Figure 2d).

Molecular dynamics simulations revealed that TNF-ligand binding influences the structural stability and dynamics of the protein. Among the three complexes, the TNF-*gallic* acid complex exhibited the highest stability, with minimal RMSD fluctuations and moderate flexibility, while the TNF-*aconitic* acid complex formed hydrogen bond interactions, contributing to its stability. In contrast, the TNF-*crocetin* complex displayed the largest structural deviations, suggesting a reduced ability to stabilize TNF conformation. These findings highlight the significance of ligand molecular properties in influencing the structural and dynamic organization of TNF, which may have implications for the design of drugs and the therapeutic targeting of TNF-related pathways.

### 2.3. In Silico ADMET Analysis of Gallic Acid, Aconitic Acid, and Crocetin: Pharmacokinetic and Toxicity Profiling

An in silico ADMET analysis was performed to evaluate the pharmacokinetic characteristics and potential interactions of *gallic* acid, *aconitic* acid, and *crocetin* with TNF. The analysis focused on absorption, distribution, metabolism, excretion, and toxicity (ADMET), key factors in assessing the potential medical use of these organic acids.

Intestinal absorption was assessed using the Caco-2 and MDCK permeability indices. The negative log(cm/s) values for Caco-2 (−5.728 to −6.055) and low MDCK permeability indices (ranging from 10^−5^ to 10^−4^ log cm/s) for all three compounds (Table 3) suggest low permeability, indicating limited passive diffusion across the intestinal barrier. The human intestinal absorption (HIA) values differed significantly, with *aconitic* acid showing the highest absorption (0.896), followed by *gallic* acid (0.085) and *crocetin* (0.015). This trend is consistent with the percentage of the absorbed compound at 20% (F20%) and 30% (F30%), with *crocetin* showing the lowest absorption and *aconitic* acid the highest. No significant inhibition or substrate activity towards P-glycoprotein (Pgp), an important protein involved in efflux mechanisms, was observed for any of the compounds, indicating their limited involvement in Pgp-mediated transport. *Crocetin* exhibited the highest plasma protein binding (92.92%), followed by *gallic* acid (53.49%) and *aconitic* acid (26.98%). High protein binding may reduce the free drug concentration, influencing its distribution in tissues.

All three compounds demonstrated low permeability across the blood-brain barrier (BBB), with logBB values less than 1. *Aconitic* acid showed the lowest permeability (logBB = 0.05), while *gallic* acid exhibited the highest (logBB = 0.099), suggesting limited penetration into the central nervous system (CNS) and minimal potential for CNS effects.

The interaction with cytochrome P450 isoenzymes (CYP) was evaluated to assess metabolic stability. *Gallic* acid and *aconitic* acid exhibited low inhibitory activity against CYP1A2, CYP2C19, CYP2C9, and CYP3A4 (IC_50_ < 0.1), indicating a low potential for interactions with these enzymes. In contrast, *crocetin* demonstrated higher inhibitory activity against CYP1A2 (0.582), CYP2C19 (0.192), CYP2C9 (0.439), and CYP2D6 (0.926), suggesting a higher potential for drug-drug interactions. *Crocetin* showed high affinity for CYP2C9 (0.996) and CYP2D6 (0.776), indicating active metabolism through these enzymes.

Clearance (CL) values differed significantly, with *gallic* acid having the highest clearance (10.108 L/h) and *crocetin* the lowest (0.744 L/h). The half-life (T_1_/_2_) of *crocetin* was the longest (0.738 h), whereas *gallic* acid had the shortest (0.947 h), reflecting differences in the rate of elimination from the body.

Inhibition of hERG channels, a marker for cardiotoxicity, was minimal for all compounds (IC_50_ < 0.05 μM), suggesting a low risk for QT interval prolongation. Hepatotoxicity (H-HT) varied, with *crocetin* exhibiting the highest risk (0.815) compared to *gallic* acid (0.433) and *aconitic* acid (0.481).

All three compounds showed low carcinogenic potential (0.016–0.406), with *crocetin* exhibiting the highest predicted carcinogenicity (0.406) and *aconitic* acid the lowest (0.016). Toxicological pathway analysis (Tox21) revealed that *crocetin* had the highest activity related to NR-PPAR-gamma (0.453) and SR-ARE (0.985), which are associated with oxidative stress and activation of nuclear receptors.

To complement the numerical data, radial (spider) plots were generated to provide a visual representation of key physicochemical properties of the compounds.

**Figure 3:**
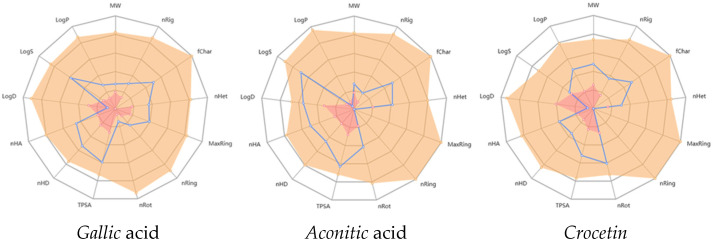
Radial Spider Plot Representation of the Physicochemical Properties of *Gallic* Acid, *Aconitic* Acid, and *Crocetin*.

The following physicochemical descriptors were analyzed: molecular weight (MW), number of rings (nRig), formal charge (fChar), number of heteroatoms (nHet), maximum ring size (MaxRing), number of rigid bonds (nRig), number of rotatable bonds (nRot), topological polar surface area (TPSA), number of hydrogen bond donors (nHD), number of hydrogen bond acceptors (nHA), n-octanol/water distribution coefficient at pH 7.4 (logD), aqueous solubility (logS), and n-octanol/water partition coefficient (logP) (Figure 3).

Beeswarm plots explain the relationship between the descriptor and the predicted value. For this, the plot uses a blue-red color scheme. In the abovementioned illustrative example, higher Crippen partition coefficient values (red-colored) cause an increase in SHAP values, while lower values (blue-colored) cause lower SHAP values.

*Gallic* acid showed the highest hydrophilicity, *crocetin* the highest lipophilicity, and *aconitic* acid was intermediate. The diagrams highlight the structural complexity of *crocetin*, its high lipophilicity, and its potential metabolic instability.

### 2.4. Influence of Organic Acids on the Expression of TNF

**Figure 4:**
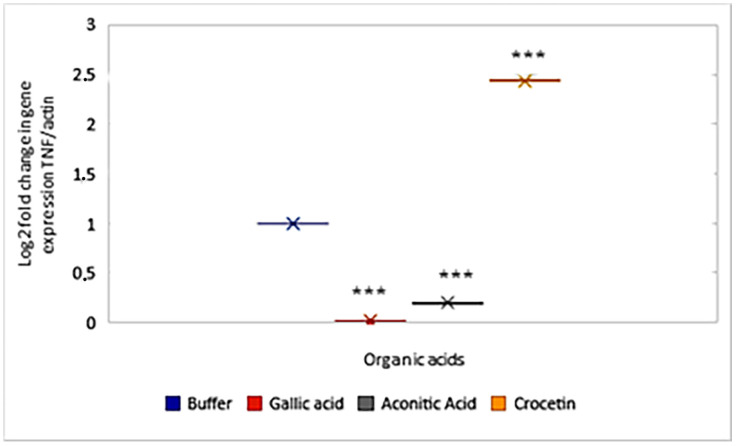
Effect of organic acids on the expression of the TNF gene. Asterisk: Standard deviation of 2^−∆∆Ct^ values, *** *p* ≤ 0.01.

A study of the ability of organic acids to bind to the TNF molecule revealed distinct properties for each compound. *Crocetin* demonstrated the highest TNF binding affinity, *gallic* acid formed the most stable protein-ligand complex, and *aconitic* acid exhibited moderate binding while maintaining key hydrogen bond interactions. These findings suggested that the compounds could be used as potential inhibitors of TNF expression. Therefore, we assessed the effect of these compounds on TNF expression in a mouse leukocyte model. The results (Figure 4) established that the studied compounds can indeed modulate gene expression.

Thus, *gallic* acid is capable of reducing gene expression by more than 10 times, *aconitic* acid by 5 times, and *crocetin*, on the contrary, increases gene expression by more than 2 times.

## 3. Discussion

Tumor necrosis factor (TNF) plays a critical role in regulating inflammatory processes, immune responses, and cellular apoptosis. Dysregulated TNF activity is associated with numerous autoimmune and inflammatory disorders, including psoriasis, inflammatory bowel disease, and rheumatoid arthritis. The identification of novel natural compounds capable of modulating TNF activity represents an important focus in contemporary biomedical research. In this study, we explored the potential of organic acids as TNF activity modulators.

**Figure 5:**
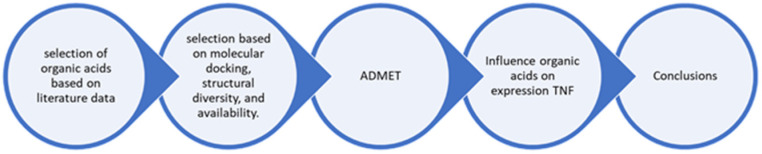
Diagram of this research.

In our studies, we used a method to assess the immunomodulatory capacity of some organic acids, as shown in Figure 5.

The immunomodulatory qualities of ten different organic acids were assessed. Their selection was based on existing literature regarding their biological characteristics [31,32,33].

Phenolic compounds, including caffeic acid and its derivatives, demonstrate significant biological activity with potential health benefits. These compounds exhibit immunomodulatory, anti-inflammatory, and antioxidant properties [31] and are widely present in dietary sources. Caffeine, a related compound, has shown therapeutic potential against metabolic syndrome and associated risk factors [32] and exhibits antiproliferative, neuroprotective, and antimicrobial effects [33]. Another notable phenolic compound, syringic acid, has been reported to possess antidiabetic, cardioprotective, and anticancer activities due to its antioxidant capacity and ability to regulate transcription factors and enzymatic activity [34].

These bioactive compounds have also been shown to enhance immune function, stimulating splenocyte proliferation and increasing cytotoxic T lymphocyte and natural killer cell activity [33,34]. Caffeic acid derivatives and similar phenolic compounds represent promising candidates for further research and potential drug development due to their wide-ranging therapeutic potential [34]. Additionally, *crocetin*, a carotenoid found in saffron, has demonstrated immunomodulatory, antioxidant, and anti-inflammatory effects [35,36,37]. It has been associated with antitumor activity, improved brain oxygenation, and enhanced oxygen diffusion [38,39]. *Crocetin* modulates immune responses by balancing Th1/Th2 and Th17/Treg subsets, reducing NF-κB activation, and suppressing nitric oxide production in human lymphocytes [39,40].

*Gallic* acid, a natural secondary metabolite found in various plant sources, also exhibits potent anti-inflammatory properties by modulating the MAPK and NF-κB signaling pathways, thereby reducing inflammatory mediator release [33]. Both *crocetin* and *gallic* acid display low toxicity and are well tolerated in experimental models, making them promising candidates for inflammatory disease treatment [33,37]. Octacosanoic acid, a long-chain saturated fatty acid derived from marine sponges, has demonstrated various biological activities [40]. Its structurally related compound, octacosanol, exhibits antifatigue, antioxidant, anti-inflammatory, and antitumor effects, as well as influencing immune function and energy metabolism through pathways such as AMPK, PI3K/Akt, and MAPK/NF-κB [40]. Similarly, octadecanoids, oxygenated derivatives of 18-carbon fatty acids, play a crucial role in inflammation, nociception, and cell proliferation across mammalian, bacterial, and fungal systems [35]. Additionally, plant-derived pentacyclic triterpenes exhibit a broad spectrum of pharmacological activities, including antioxidant, anticancer, and anti-inflammatory effects, which are believed to be mediated by immune system modulation [30].

Following computational screening, three promising candidates were selected for in-depth analysis. Molecular docking studies revealed that *crocetin* exhibited the highest binding affinity to TNF, with binding energy values ranging from −5.6 to −4.6 kcal/mol. The TNF-*crocetin* complex formed multiple hydrogen bonds and stable van der Waals interactions, suggesting ligand retention within the active site of the protein. Given *crocetin*’s natural origin and well-documented pharmacological properties, it holds potential as a TNF inhibitor and warrants further investigation as a candidate for anti-inflammatory and antioxidant drug development. *Aconitic* acid displayed the lowest binding affinity, with energy values ranging from −3.9 to −3.5 kcal/mol. Nevertheless, hydrogen bonding analysis revealed stable interactions with key residues Asn125, Leu105, and Val214, indicating that it may serve as a scaffold for structural modifications aimed at enhancing TNF binding. *Gallic* acid, included as a reference compound, exhibited stable interactions with TNF, forming hydrogen bonds with His99, Gly226, and Ala112, with binding energy values ranging from −4.7 to −4.2 kcal/mol. While slightly less effective than *crocetin*, *gallic* acid’s well-characterized biological activity and high solubility make it a valuable subject for further pharmacological studies [39].

Molecular dynamics simulations provided additional insights into the structural stability of TNF complexes in an aqueous environment under physiological conditions. Among the ligands investigated, *gallic* acid formed the most stable complex, making it a strong candidate for further study. *Crocetin* exhibited the highest TNF affinity but induced significant conformational changes in the protein, which may impact TNF structural dynamics. *Aconitic* acid, despite lower stability, maintained hydrogen bonding interactions, suggesting its potential for chemical modifications to enhance its binding properties.

These findings suggest that *crocetin* has the greatest potential as a TNF inhibitor, surpassing *gallic* acid in binding efficiency and complex stability. While *gallic* acid demonstrated slightly lower affinity, its well-documented biological activity supports its continued investigation as a reference compound in TNF-targeting studies. Although *aconitic* acid exhibited weaker TNF binding, its stable interactions with key amino acid residues highlight its potential as a lead structure for future chemical modifications to enhance inhibitory activity.

ADMET analysis further highlighted the pharmacokinetic differences among the studied organic acids, providing insight into their potential as TNF modulators. *Crocetin* displayed high plasma protein binding and moderate metabolic stability but also exhibited risks of hepatotoxicity and drug interactions. *Aconitic* acid showed high absorption but low bioavailability, whereas *gallic* acid exhibited rapid clearance, which may limit its therapeutic applications. Analysis of their structures within the ADMET framework revealed that the compounds possess several qualities typical of medicinal preparations, including lipophilicity, metabolic stability, and oral bioavailability. These characteristics of the investigated organic acids demonstrate their potential efficacy as immunomodulators and emphasize the necessity for further research aimed at optimizing the chemical structures of these compounds to enhance their pharmacokinetic profiles and minimize potential toxicity [33,36,41].

A study on the effect of *gallic*, *aconitic*, and *crocetin* on the expression of TNF showed that these organic acids are indeed capable of modulating its expression level. It was demonstrated that while *gallic* and *aconitic* acids are pronounced inhibitors of gene expression, crocetin, in contrast, stimulates cytokine synthesis. This opposing effect may be attributed to the different mechanisms by which the compounds interact with the TNF protein or its regulatory pathways.

Interestingly, although molecular docking showed *crocetin* to have the highest predicted affinity for TNF (−5.6 to −4.6 kcal/mol), *in vivo* experiments revealed a paradoxical increase in TNF gene expression. This apparent contradiction may be due to a number of factors.

First, *crocetin* may act as a ligand-dependent partial agonist of TNF or its receptors, stabilizing the active conformational state of the protein rather than inhibiting signaling activity. Similar behavior has been described for small molecules that bind to TNFR1 and TNFR2 receptors, which do not suppress but instead enhance cytokine production. [42,43].

Second, the interaction of *crocetin* with TNF may be non-inhibitory or allosteric, altering the protein conformation or signal transduction pathway without direct blockade of the active site. This is supported by molecular dynamics results that revealed significant conformational changes in the TNF-*crocetin* complex, including higher RMSD and SASA values compared to other acids. Such changes are more likely to indicate structural destabilization, which may lead to exposure of interaction surfaces or promote the formation of signaling complexes.

Third, *crocetin* is known to influence several immune signaling pathways, including NF-κB and PPAR-γ [44], which may indirectly activate TNF expression at the transcriptional level. *In vivo* effects occur within complex regulatory networks, where *crocetin* may act indirectly or synergistically with other mediators of the immune response.

Thus, despite the high predicted binding of *crocetin* to TNF based on in silico data, its actual biological effect appears to depend on receptor subtype selectivity, protein conformational dynamics, and systemic immune context. These data highlight the need to combine computational modeling with experimental validation to fully understand the mechanisms of action of potential TNF regulators under physiological conditions.

*Crocetin*, having a high ability to bind with the protein molecule without creating strong complexes, stimulates gene expression, and *gallic* and *aconitic* acids, forming hydrogen bonds with the protein molecule, inhibit gene expression. This behavior of organic acids can be used to create new immunomodulatory drugs.

One of the key findings of this study is the identification of a novel mechanism of action for organic acids. Traditionally, these compounds were thought to influence cellular processes primarily through ion dissociation and ATP inhibition. However, our results demonstrate their direct interaction with TNF, offering new insights into their biological activity and expanding our understanding of inflammation regulation. These findings provide a foundation for future research into pharmaceuticals that selectively modulate TNF activity, offering potential therapeutic strategies for inflammatory, oncological, and autoimmune diseases [26,37,38].

## 4. Materials and Methods

### 4.1. Materials

Organic acids were purchased from the Merck company (*Gallic* acid (G7384, Merck, Darmstadt, Germany), *Syringic* acid (S6881 Merck, Darmstadt, Germany), *Caffeic* acid (C0625, Merck, Darmstadt, Germany), *Aconitic* acid (A3412, Merck, Darmstadt, Germany), *Crocetin* (SML3255, Merck, Darmstadt, Germany), *Octacosanoic* acid (284432, Merck, Darmstadt, Germany), *Pentatriacontanoic* acid (8.00661, Merck, Darmstadt, Germany), *Ferulic* acid (Y0001013, Merck, Darmstadt, Germany), *Vanillic* acid (H36001, Merck, Darmstadt, Germany), *Isocitric* acid (H36001, Merck, Darmstadt, Germany)). Their 2D and 3D molecular structures were obtained from *PubChem* [42] and *ChemSpider* [43]. The 3D structure of murine tumor necrosis factor (TNF, UniProt ID: P06804) was retrieved from the *AlphaFold* database, and additional protein structures were predicted using *AlphaFold* or *OmegaFold* [44,45].

### 4.2. Methods

#### 4.2.1. Molecular Docking

Molecular docking was performed using the software suites *AutoDock Vina* 1.1.2 (http://vina.scripps.edu (accessed on 1 June 2025)) and *AutoDockTools* (http://mgltools.scripps.edu/downloads (accessed on 1 June 2025)) to assess ligand binding to tumor necrosis factor (TNF) [46]. The 3D structure of murine TNF-α was obtained from the *AlphaFold Protein Structure Database* (UniProt ID: P06804). As no crystal structure was available for the mouse TNF-ligand complex, the AlphaFold-predicted structure was validated for use in docking and MD simulations.

Ligand structures were retrieved from PubChem and converted to 3D using *Open Babel.* All ligands and the receptor were energy-minimized using the MMFF94 force field. Hydrogen atoms and Gasteiger charges were added using *AutoDock Tools*. The docking grid was centered on the predicted ligand-binding site based on *AlphaFold* residue confidence (pLDDT > 90), with a box size of 30 × 30 × 30 Å and a grid spacing of 1.0 Å.

To validate the docking protocol, a redocking test was performed using a known TNF-binding small molecule (from PDB ID: 2AZ5) to confirm that the predicted binding pose aligned with the experimental binding site (RMSD < 2.0 Å).

#### 4.2.2. ADMET Analysis

The pharmacokinetic properties of the studied compounds, including absorption, distribution, metabolism, excretion, and toxicity (ADMET), were evaluated using the online platform *ADMETlab 2.0* (https://admetmesh.scbdd.com (accessed on 1 June 2025)) [47]. The analysis considered key parameters such as blood-brain barrier permeability, plasma protein binding, potential hepatotoxicity, and CYP450 enzyme inhibition.

#### 4.2.3. Molecular Dynamics Simulation

MD simulations were conducted using the GROMACS 2022 package with the AMBER99SB-ILDN force field for proteins and GAFF for ligands. Ligand topology files were generated using ACPYPE (https://www.bio2byte.be/acpype (accessed on 1 June 2025)) [48]. Each TNF-ligand complex was solvated in a TIP3P water box with a 1.0 nm buffer and neutralized with Na^+^/Cl^−^ ions.

The ligands were geometrically optimized using the MMFF94 force field in Open Babel. This procedure enables the attainment of the most stable conformation of the molecule, considering bond lengths, angles, torsional stresses, and steric effects.

The TNF protein, downloaded from the *AlphaFold* database, was prepared using AutoDock Tools. The procedure included the removal of water molecules and non-standard residues, the addition of polar hydrogen atoms, the assignment of Gasteiger charges, and conversion of the structure to the PDBQT format required for *AutoDock Vina*.

These steps are standard in molecular docking and ensure proper electrostatic and spatial compatibility between the ligand and the protein during the binding modeling process.

Following energy minimization, the system underwent equilibration in NVT (100 ps) and NPT (100 ps) ensembles at 300 K and 1 atm, respectively, using the Berendsen thermostat and Parrinello–Rahman barostat. Harmonic position restraints were applied to the protein during equilibration. Production simulations were run for 100 ns with a time step of 2 fs, using PME for long-range electrostatics and LINCS for bond constraints. Ligand concentrations were modeled at 150 µg/mouse equivalents (~1 mM), consistent with the *in vivo* dosing protocol.

Trajectory analyses included RMSD, RMSF, Rg, SASA, and hydrogen bonding using built-in *GROMACS* tools [49].

### 4.3. Animal Experiments

Specific pathogen-free, male BALB/c mice, aged 4–5 weeks, were used in this study. The mice were acclimatized under vivarium conditions for 2 weeks prior to the experiment. They were housed in individually ventilated cages (six mice per cage) under a 12-h light/dark cycle at a temperature of 20–25 °C. Standard rodent food and water were provided ad libitum. Each experimental group consisted of 6 animals.

Organic acids or control samples (sterile PBS, pH 7.4) via intraperitoneal injection (200 µL per mouse). The dose of organic acid was 150 µg per mouse and was administered separately in each group of mice.

Three days following preparation administration, all mice were euthanized using CO_2_ inhalation, following AVMA guidelines. Peritoneal leukocytes were collected by washing the peritoneal cavity with Dulbecco’s Phosphate-Buffered Saline (DPBS) lacking calcium and magnesium. The resulting cell suspensions were centrifuged at 1000× *g*, and the supernatant was discarded. The cell pellet was resuspended in PBS to a concentration of 2 × 10^6^ cells/mL, aliquoted, and stored frozen.

All experimental procedures with mice were approved by the Research and Production Center for Microbiology and Virology Institutional Animal Care and Use Committee (conclusion of the bioethics commission dated 4 October 2021).

### 4.4. Isolation of RNA from Peritoneal Leukocytes

Total RNA was extracted from peritoneal leukocytes using the RNeasy Mini Kit (QIAGEN, Hilden, Germany), following all manufacturer’s instructions. RNA yield (average: 182.7 ± 37.5 ng/µL) and purity (average A260/A280 = 2.1 ± 0.05) were determined using spectrophotometry (Infinite 200 Pro, Tecan Group Ltd., Männedorf, Switzerland).

### 4.5. Reverse Transcription

For each sample, 400 ng of total RNA in a final volume of 8 µL was reverse transcribed into cDNA using TaqMan^®^ Reverse Transcription Reagents (ThermoFisher Scientific, Waltham, MA, USA). The 20 µL reaction mixture contained 1.6 µL DEPC-treated water, 2 µL 10X RT Buffer, 1.4 µL 25 mM MgCl_2_, 4 µL 10 mM dNTP mix (2.5 mM each), 1 µL RNase Inhibitor (20 U/µL), 1 µL MultiScribe™ RT (50 U/µL), 1 µL 50 µM Oligo d(T)_16_, and 8 µL Template RNA. The procedure followed the manufacturer’s description. Following cDNA synthesis, the volume was adjusted to 80 µL by adding 60 µL of sterile Milli-Q water, yielding a working cDNA concentration equivalent to 5 ng/µL total RNA. The reaction protocol involved heating the RNA/Oligo d(T)_16_ mixture at 65 °C for 5 min, cooling at 4 °C for 2 min, adding the remaining reagents, and incubating at 37 °C for 30 min, followed by inactivation at 95 °C for 5 min.

### 4.6. Determination of Gene Expression Levels

Quantitative PCR (qPCR) was conducted on a QuantStudio 5 Real-Time PCR System (ThermoFisher Scientific, Waltham, MA, USA). TNF mRNA expression (Mm00443258_m1) was normalized to β-actin (Mm02619580_g1), and relative quantification was calculated by the 2^−ΔΔCt^ method [49]. Each sample was analyzed in technical duplicates, and Ct values were averaged. The final results are expressed as fold changes relative to the PBS-treated control group.

Data analysis was performed using QuantStudio Design and Analysis Software v1.4.1. Relative quantities (RQ) were calculated using the ΔΔCt method [49].

### 4.7. Statistical Analysis

Data processing and graphical representation were performed using Microsoft Excel. A one-way ANOVA followed by Fisher’s LSD post-hoc test was used to identify significant differences between experimental and control groups. All values are expressed as the mean ± standard deviation (SD). A *p*-value < 0.05 was considered statistically significant.

## 5. Conclusions

This study employed an integrated in silico and *in vivo* approach to evaluate organic acids as potential modulators of Tumor Necrosis Factor (TNF). Computational methods, including molecular docking, ADMET profiling, and molecular dynamics simulations, identified promising candidates with favorable binding interactions and dynamic stability. *Crocetin* demonstrated the strongest predicted binding affinity to TNF, while *gallic* acid formed the most stable protein–ligand complex during simulations. Although *aconitic* acid showed moderate binding affinity, it formed robust hydrogen bonds, indicating its potential as a lead structure. However, ADMET analyses revealed potential pharmacokinetic challenges, such as predicted low permeability for all three and metabolic concerns for *crocetin*, underscoring the need for compound optimization.

These findings highlight both the potential and the complexities of using organic acids as TNF modulators. Crucially, the *in vivo* gene expression experiments revealed that while *gallic* and *aconitic* acids inhibited TNF synthesis, *crocetin* exerted a stimulating effect. This contrasts with its strong in silico binding predictions, underscoring the necessity of experimental validation and suggesting that computational models must be complemented by biological assays to capture the full spectrum of activity, which may involve differing interaction mechanisms or downstream pathways. Despite potential pharmacokinetic hurdles, this study provides a crucial foundation. Future research should focus on validating these findings through *in vitro* and *in vivo* functional assays, elucidating the paradoxical mechanism of *crocetin*, and exploring structural modifications to optimize the therapeutic potential of candidates like *gallic* and *aconitic* acids for TNF-mediated diseases.

## Figures and Tables

**Table 1:** Binding Affinity of Selected Ligands with TNF Protein.

No.	Compound	Structure	3D Structure	Binding Affinity, (kcal/mol)	
				Max	Min
1	*Gallic* acid	C_7_H_6_O_5_	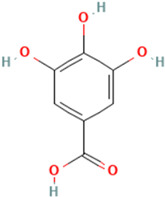	−4.7	−4.2
2	*Syringic* acid	C_9_H_10_O_5_	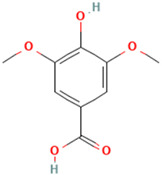	−5.2	−3.8
3	*Caffeic* acid	C_9_H_8_O_4_	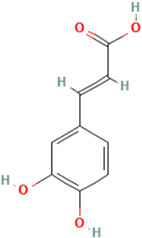	−4.8	−3.9
4	*Aconitic* acid	C_6_H_6_O_6_	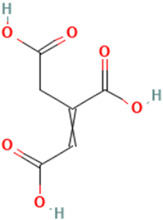	−3.9	−3.5
5	*Crocetin*	C_20_H_24_O_4_	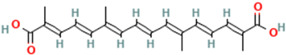	−5.6	−4.6
6	*Octacosanoic* acid	C_28_H_56_O_2_	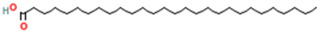	−3.2	−2.6
7	*Pentatriacontanoic* acid	C_35_H_70_O_2_	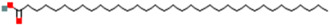	−3.2	−2.7
8	*Ferulic* acid	C_10_H_10_O_4_	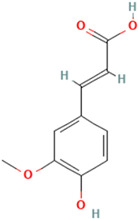	−4.8	−4.0
9	*Vanillic* acid	C_8_H_8_O_4_	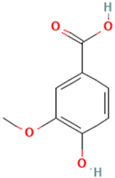	−5.0	−3.8
10	*Isocitric* acid	C_6_H_8_O_7_	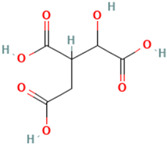	−4.3	−3.7
11	*Curcumin*	C_21_H_20_O_6_	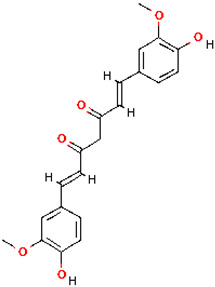	−4.9	−4.7
12	*Thymoquinone*	C_10_H_12_O_2_	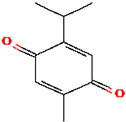	−4.5	−4.0

**Table 2:** Interaction Features of TNF Protein with Ligands.

No.	Compound	H-Bonds	Van Der Waals Force	2D Visualization in LigPlot
1	*Gallic* acid	His99, Gly226, Ala112	Ser225, Val96, Arg111	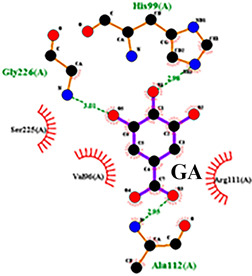
2	*Aconitic* acid	Asn125, Leu105, Val214	Glu213, Gln104, Pro217	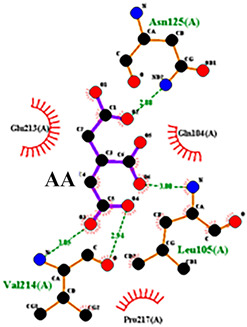
3	*Crocetin*	Tyr197	Tyr138, Tyr229, His94, Gly226, Ala112, Val96, Ser225, His99, Arg111	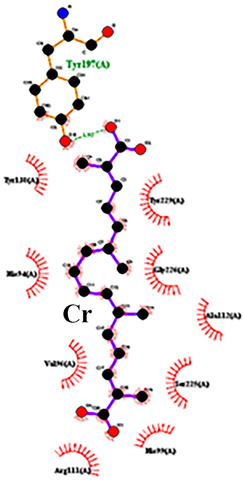

**Table 3:** Absorption, Distribution < Metabolism, Excretion, and Toxicity of Organic Acids.

Property	Model Name	GA	AA	Cr	Unit
Absorption	Caco-2 Permeability	−5.728	−6.055	−5.511	Numeric (log Papp in 10^−6^ cm/s)
	HIA	0.085	0.896	0.015	Probability (0–1)
Distribution	PPB	53.49	26.98	92.92	%
	BBB permeability	0.099	0.05	0.083	Numeric (log BB)
Metabolism	CYP2C9 substrate	0.061	0.192	0.996	Probability (0–1)
	CYP2D6 inhibitor	0.008	0.018	0.776	Probability (0–1)
Excretion	CL (mL/min/kg)	10.108	1.776	0.744	Numeric (mL/min/kg)
	T_1/2_	0.947	0.924	0.738	Numeric (h)
Toxicity	hERG Blockers	0.017	0.002	0.005	Probability (0–1)
	H-HT	0.433	0.481	0.815	Probability (0–1)
	Carcinogenicity	0.024	0.016	0.406	Probability (0–1)

## Data Availability

All the data generated or analyzed during this study are included in the published article.

## Abbreviations

The following abbreviations are used in this manuscript:

TNF	Tumor necrosis factor
ADMET	Absorption, distribution, metabolism, excretion, and toxicity
MD	Molecular dynamic
